# Glances at the Hospitals

**Published:** 1902-09-20

**Authors:** 


### GLANCES AT THE HOSPITALS.

### KILMARNOCK INFIRMARY.

THE total ordinary income during the year was £4,550, and the ordinary expenditure was £4,950, while the general subscriptions were £43 less than in the previous year, the workpeople's own subscriptions were £9 higher. The ordinary expenditure has considerably increased with rise in prices of various articles of consumption, and the governors strongly appeal to the general public to increase their liberality so that the hospital may be efficiently maintained. In addition to this the surgeons are hampered by the insufficient size of the operation room, and to enlarge and equip this will necessitate the expenditure of £1,000. Kilmarnock contains 30,000 inhabitants, and it would be strange if 30 could not be found who are able and willing to subscribe £30 each for the new work needed in the operation theatre. At the beginning of the year the hospital contained 126 patients, and 1,093 were admitted during the twelve months, making a total of 1,119. Of these 797 were cured, 143 improved, 52 not improved, 3 were dismissed, and 99 died; 133 were left in residence. The number of fever cases admitted was 287, and the average residence of those treated to a termination was 44 days. Of the 363 medical cases the average residence was 31 days, and of the 443 surgical cases the average residence was 30 days. Of scarlet fever alone there were 193 cases with 11 deaths, and 113 cases of enteric fever had 14 deaths. Of 35 cases of pneumonia, 27 recovered, 2 were improved, 1 not improved, 2 died, and 3 were under treatment. All the statistics are carefully prepared and the information clearly given. We should like to see a table of anæsthetics used during the operations. Chloroform is by far the most common agent in use north of the Tweed, and it would be interesting to have the exact figures for comparison with English hospitals.

### ROYAL LONDON OPHTHALMIC HOSPITAL.

WE are informed that the year began with a deficiency of £2,591, and that during the year there was an excess of expenditure over income of £2,577, making a total deficiency of over £5,000. The result of this was that 68 beds remained unopened for want of funds, and a loan had to be obtained to keep the hospital open. Fortunately, a special donation was made from King Edward's Hospital Fund, and it has been found practicable to open 18 additional beds. The committee finish their report by an earnest appeal for £5,169 to clear the hospital from its debt, and £8,000 in further annual subscriptions or donations for the current expenses. The ground rent amounts to £1,210 a year, and donations to meet this sum are also asked for. Unquestionably the hospital is in sore straits; and when it is stated that there were 1,866 in-patients and 31,258 out-patients treated during the year, there cannot be a doubt of the deserving nature of the institution. It may be mentioned that the operation for cataract was performed 672 times, and these may be taken as an index of the amount of misery relieved by the hospital.

### BEDFORD COUNTY HOSPITAL.

THE total income of the hospital was £4,866, and the current expenditure was £4,966. Appeals were made on behalf of the hospital by the Governors, and the result was fairly satisfactory as £162 were obtained from new subscribers; £124 from increased subscriptions; and £110 from special donations. Of course these sums must be to some extent discounted by the losses from death, withdrawals and removals; and unfortunately there is a balance due to the treasurer of £1,307. On January 1st the hospital contained 48 patients, and 873 were admitted since, making a total of 921 under treatment. Of these 511 were discharged cured, 217 relieved, 53 not relieved, and 57 died—of the latter 14 died on the day of admission; 83 patients remained in the hospital on December 31st. The average daily number was 65, and the average stay in the hospital was 25 days. The average cost of each in-patient was £4 19s. 6d., and the average cost per bed occupied was £70 10s. The weekly cost for provisions per patient was 5s. 5d. The average daily number of the household was 40, and the cost of provisions was 6s. per head. This is one of the newest county infirmaries in England, and including the Nurses' Home it has cost £39,800, of which sum £6,400 remains unpaid. It is a pity that this debt could not be wiped off so that the institution could continue and extend its career of usefulness. £6,000 is not a large sum to raise, and we commend the matter to some energetic and influential inhabitant of the county. The Roentgen Ray apparatus was presented to the hospital by the Bedfordshire Medical Society, and during the year it was used in 134 cases; 98 of these being fractures or dislocations; 26 were for the detection of foreign bodies; 6 for examination of the chest; 3 for stone, and one case of lupus was treated. The medical and surgical tables are properly compiled, and the anæsthetic used is stated in every one of the operations, of which there were 460.

### ROYAL MINERAL WATER HOSPITAL, BATH.

THIS infirmary has a wide range for its usefulness as it admits patients from any part of Great Britain or Ireland, and the patients are gratuitously supplied with medical advice, food, washing and nursing; and it goes without saying that subscribers to the charity should be found in every part of the country. Nevertheless, we find that the annual subscriptions from private persons amounted only to £576, a sum ridiculously low when all things are taken into consideration. Additions have lately been completed, and the buildings can now accommodate 165 patients. These additions have cost £6,000, and the result of a special appeal to meet this cost resulted in only £509, a condition of things hardly creditable to the population at large. Nearly £5,000 has had to be withdrawn from capital to meet the payments due to contractors, and this withdrawal will materially reduce the annual income unless something is done to improve it. The hospital only contained 48 patients at the beginning of the year, and 864 were admitted during the year, making a total of 912. The diseases under treatment were rheumatism, rheumatoid arthritis, sciatica, gout, paralysis, neuritis, lead poisoning, eczema, psoriasis and chorea. Of the total number 13 were cured, 672 were relieved, 1 died and the rest were either unsuitable cases or were not relieved. The hospital existed as far back as 1742, and since that year the large number of 77,257 cases have been treated in it.

